# Racial and Ethnic Differences in Preventive Services Use After the Affordable Care Act’s Enhancement of Medicare Benefits

**DOI:** 10.1093/geroni/igaa057.324

**Published:** 2020-12-16

**Authors:** Mohammad Usama Toseef, Wassim Tarraf

**Affiliations:** 1 Wayne State University, Farmington Hills, Michigan, United States; 2 Wayne State University, Detroit, Michigan, United States

## Abstract

As a consequence of the Affordable Care Act’s enhancements of Medicare benefits, certain recommended clinical preventive services became available to Medicare beneficiaries without cost-sharing. We study the impact of these mandates on racial/ethnic disparities in the use of preventive services among traditional Medicare beneficiaries. We analyze nationally representative data on non-institutionalized Medicare seniors from the 2006-2016 Medical Expenditure Panel Survey (N=27,124). Our preventive services of interest include yearly receipt of cholesterol check, blood pressure test, flu shot, endoscopy, blood stool test, clinical breast examination, mammography and prostate exam. We estimate propensity score weighted difference-in-difference (DID) models to test for differences in preventive services utilization by race/ethnicity. Among traditional Medicare beneficiaries, we do not observe significant change in the use of most preventive services for Blacks and Hispanics compared to their White counterparts. However, Hispanics have significantly increased their use of blood stool tests relative to whites. Overall, we do not find major evidence to support a differential effect of reforms on race/ethnic minorities’ uptake of preventive services following the mandates. Our results suggest that despite an overall benefit trough services expansion and cost-sharing elimination race/ethnic group differences persist. As such, disparities might continue and would require additional interventions. Reduction in disparities is a stated goal of US policy for many decades and achieving equity might require additional work and more varied and targeted interventions.

